# Computational insights into magnetoelectric nanoparticles for neural stimulation

**DOI:** 10.3389/fnins.2025.1583152

**Published:** 2025-04-28

**Authors:** Alessia Vezzoni, Emma Chiaramello, Valentina Galletta, Marta Bonato, Marta Parazzini, Serena Fiocchi

**Affiliations:** ^1^Multi-Scale Robotics Lab, Institute of Robotics and Intelligent Systems, ETH Zurich, Zurich, Switzerland; ^2^Institute of Electronics, Computer and Telecommunication Engineering (IEIIT), National Research Council (CNR), Milan, Italy; ^3^Department of Electronics, Information and Bioengineering (DEIB), Politecnico di Milano, Milan, Italy

**Keywords:** magnetoelectric nanoparticles, neural stimulation, numerical methods, computational neuroscience, neuroengineering

## Abstract

**Introduction:**

This study investigates the potential of magnetoelectric nanoparticles (MENPs) as a novel tool for localized electric stimulation of the central nervous system at single-neuron level, addressing the need for precise and minimally invasive neural modulation.

**Methods:**

Using a computational framework based on finite element methods coupled with neuronal dynamics simulations on a realistic model of a hippocampal CA1 pyramidal neuron, the study evaluates how MENPs' stimulation parameters influence neural activation. Analyses included electric potential distributions, the activating function along the axon, amplification coefficients required for action potential generation, spike propagation, and membrane potential. The study initially focused on highly localized stimulation using a nanometric MENP close to the axon and then demonstrated the feasibility of a more realistic framework involving a micrometric cluster of MENPs. To emulate physiological signal convergence, the summation effects of multiple MENPs strategically positioned across the basal dendritic tree near the axon were explored.

**Results and discussion:**

The findings revealed the critical role of MENPs' configuration, location, and modulating stimuli in shaping neuronal responses, highlighting the feasibility of MENPs as a cutting-edge approach for precise neural stimulation. This work provides a foundation for integrating MENPs into therapeutic strategies for neurodegenerative diseases.

## 1 Introduction

The brain functioning is governed by interconnected neurons communicating through a complex network of chemical and electrical signals, with synapses between axons and dendrites enabling information transfer via electric charges, neurotransmitters, and action potentials (AP). The resulting electrical activity, driven by hyperpolarization and depolarization, occurs both spontaneously and in response to external stimuli, allowing for the control of localized brain functions through electrical stimulation (Pardo and Khizroev, 2022). Disturbances within this collective structure led to neurodegenerative diseases, affecting cortical, and deep brain regions (Kujawska and Kaushik, 2023).

Alzheimer's disease (AD) stands as the primary neurodegenerative disorder, characterized by progressive cognitive decline and memory impairment, notably linked to the neural activity in the hippocampus CA1 region (Alzheimer's Disease and Dementia, 2025; Scheff et al., 2007; Zarifkar et al., 2024). Currently, no cures can halt or reverse its progression, prompting researchers to investigate non-drug therapies to prevent or slow memory loss (Hescham et al., 2013). To this extent, electrical stimulation for the modulation of communication among neurons' circuitry has proven effective in AD. For example, transcranial direct current stimulation (tDCS), deep brain stimulation (DBS), and temporal interference (TI) have shown promise in addressing AD symptoms by modulating neuronal activity in the CA1 subfield of the hippocampus that supports memory function (Zarifkar et al., 2024; Bjekić et al., 2021; Violante et al., 2023; Hescham et al., 2020; Mankin and Fried, 2020; Hescham et al., 2015). However, these techniques are hindered by limitations, such as scarce penetration, low spatial resolution, invasiveness, and inconsistent spatial selectivity, highlighting the need for innovative stimulation methods (Violante et al., 2023; Alosaimi et al., 2023; Matsumoto and Ugawa, 2017; Sparing and Mottaghy, 2008).

In recent years, nanotechnology has emerged as a compelling novel approach for the treatment of neurodegenerative diseases, since the dimensions of nanoengineered materials allow their structures to engage with neuronal networks at the level of single cells, offering these materials the capability to bring about unprecedented outcomes in biological systems (Bayda et al., 2019). In this context, attaining a wireless, high-resolution, and minimally invasive transmission of signals to nanometric injectable devices could achieve groundbreaking advancements in neural stimulation techniques.

To address this challenge, in recent years, solutions for coupling magnetic and electric signals, related to the magnetoelectric (ME) effect, have been explored, offering a promising alternative to electrode-based technologies by reducing invasiveness and enabling focused stimulation (Alrashdan et al., 2024).

A ME material is a generic term used to describe a substance that, due to its chemical composition, demonstrates linear interdependence between magnetic and electronic characteristics (Kargol et al., 2012). The ME effect is characterized by alterations in the electric polarization of a material when subjected to a magnetic field—termed the direct effect—or changes in its magnetization when exposed to an electric field—referred to as the converse effect (Kopyl et al., 2021).

The ME coupling can emerge either from a direct interaction between ferromagnetic and ferroelectric phases, as in the case of single phase multiferroics, or indirectly through strain (Eerenstein et al., 2006). Within the last-mentioned category, noteworthy attention is directed toward artificially engineered composite materials that incorporate ferromagnetic and piezoelectric phases, since these materials are attractive for many applications due to their superior ME parameters at room temperature (Kargol et al., 2012). The conversion of magnetic energy into electric energy leverages the piezomagnetic (or magnetostrictive) properties of the ferromagnetic phase and the piezoelectric properties of the ferroelectric phase. Magnetostrictive stress, generated in the magnetic phase by the variation of an applied magnetic field ΔH, is transmitted through the interface between the ferroic phase to the ferroelectric phase. This, in turn, induces a change in polarization ΔP and an associated electric field ΔE due to the piezoelectric effect (Kargol et al., 2012). The ME coefficient α serves as the key metric characterizing the performance of multiferroic composites. In its simplest form, α is defined as the ratio of the change in polarization in response to the change in the magnetic field: α = ΔP/ΔH. Alternatively, the ME voltage coefficient αE = ΔE/ΔH can be employed (Kargol et al., 2012).

Multiferroic structures, in the most widely used configuration of magnetoelectric nanoparticles (MENPs) composed of a cobalt ferrite (CoFe_2_O_4_) ferromagnetic core and a barium titanate (BaTiO_3_) ferroelectric shell, have recently garnered attention as innovative promising neural stimulators, that can be guided to cross the blood-brain barrier (BBB) into the brain area to be stimulated, and activated wirelessly via external magnetic field gradients (Guduru et al., 2015). MENPs, indeed, act as an extremely efficient transducer of the magnetic field, biocompatible in a wide range of frequencies and magnitudes, in the electric field, the prime mover of the modulation of neuronal activity. Consequently, MENPs could achieve extremely promising therapeutic potential in the field of electric neuromodulation techniques.

The use of MENPs for brain stimulation, first suggested by Yue et al. (2012) through computational studies, has been validated with *in vitro, in vivo* and *ex vivo* experiments (Guduru et al., 2015; Zhang et al., 2022, 2024; Nguyen et al., 2021; Kozielski et al., 2021), while showing strong biocompatibility (Nguyen et al., 2021; Kozielski et al., 2021) and controllable clearing rates (Hadjikhani et al., 2017), researchers demonstrated that MENPs, delivered to the desired site via intranasal, intravenous, or stereotactic administration, guided using a magnetic field gradient, and activated by applying a DC magnetic field and/or an AC magnetic field at low frequency, can effectively induce and modulate neural activity, restore healthy electrical patterns, and influence animal behavior (Guduru et al., 2015; Zhang et al., 2022, 2024; Nguyen et al., 2021; Kozielski et al., 2021).

In addition to these encouraging experimental findings, computational modeling serves as an essential tool in advancing the development of next-generation neuromodulation devices, by reducing the need for animal experiments and related costs, while accurately predicting physiological outcomes to speed up validation and translation to applications (Pratiwi et al., 2022).

In computational neuroengineering, the modeling usually integrates electromagnetic (EM) fields distributions, elicited by stimulating sources, with biophysical models to evaluate neural responses (Romeni et al., 2020; Paffi et al., 2013). The EM distribution is typically solved using finite element modeling (FEM), a numerical approach for solving partial differential equations (Romeni et al., 2020). Computational methods are essential for determining solutions and evaluating electrical properties within the brain tissue. Concurrently, advances in neural modeling have provided realistic neuron models for precise estimation of induced EM field effects (Neufeld et al., 2016; McNeal, 1976; Rattay, 1986, 1998).

In the outlined context, this work delves into investigating, through computational approaches, the capacity of MENPs to electrically stimulate a pyramidal neuron from the hippocampus CA1 region, with the aim of uncovering novel insights into their potential for targeted neuromodulation. To address the lack of studies on the local interaction between MENPs and neurons at the single particle-single neuron level, the initial focus of this study was on the stimulation produced by a nanometric MENP placed close to the axon. However, in practical applications, a solution containing a specific concentration of nanoparticles is delivered to the target region, rather than individual MENPs. Under the influence of external fields, the nanoparticles align along the direction of the magnetic field. Assuming the presence of a large number of MENPs occupying a certain volume, with dimensions depending on the concentration of the injected solution (Kozielski et al., 2021), it is possible to approximate the distribution electric field in the surroundings as the one due to a macroscopic dipolar distribution. An investigation into the stimulation effects due to this condition was considered in the study. Moreover, under physiological conditions, neurons process a vast array of synaptic inputs that are widely distributed across their structure (Magee, 2000). Consequently, a central goal in neuroscience is to unravel how excitatory and inhibitory signals converge within the dendritic tree to produce APs at the axon level (Müller and Remy, 2013). To replicate this characteristic with the innovative stimulation method represented by MENPs, the scenario in which multiple individual MENPs are strategically positioned at various locations around the basal dendritic arbor—associated with synaptic failure in AD (Selkoe, 2002)—and their combined influence leads to spatial and temporal summation effects, was modeled and analyzed. Overall, the findings establish the feasibility of using MENPs as a cutting-edge and precise tool for neural stimulation, highlighting critical factors, such as configuration and modulating stimuli, that influence neuronal activation. These results provide a foundational framework for the development of MENPs-based strategies, with potential applications in the treatment of neurodegenerative diseases and other disorders requiring targeted neural modulation.

In summary, the effects of different stimulation parameters, namely MENPs' configuration (concentration, orientation, and distance), modulating pulse frequency and amplitude, and neuronal targets, were evaluated in terms of electric distributions, amplitude thresholds of stimuli needed to trigger APs, axon's membrane potential, and summation effects across the dendrites.

## 2 Materials and methods

In this section, first the MENP model and the EM simulation parameters are outlined; subsequently, the neuron model and the neuronal dynamics settings are presented; finally, the different simulation settings are depicted, along with the metrics employed in the analysis of the results. The Sim4Life (ZMT Zurich MedTech AG, Switzerland) simulation platform^1^ was used for the purpose of this study. Among many solvers, Sim4Life includes the neuronal solver, which combines Multiphysics FEM and neural simulation optimized software with human and animal validated neuronal models and is thus well suited for the solution of computational neuroengineering problems (Romeni et al., 2020; Neufeld et al., 2016).

### 2.1 MENP model and EM simulation settings

**Figure 1:**
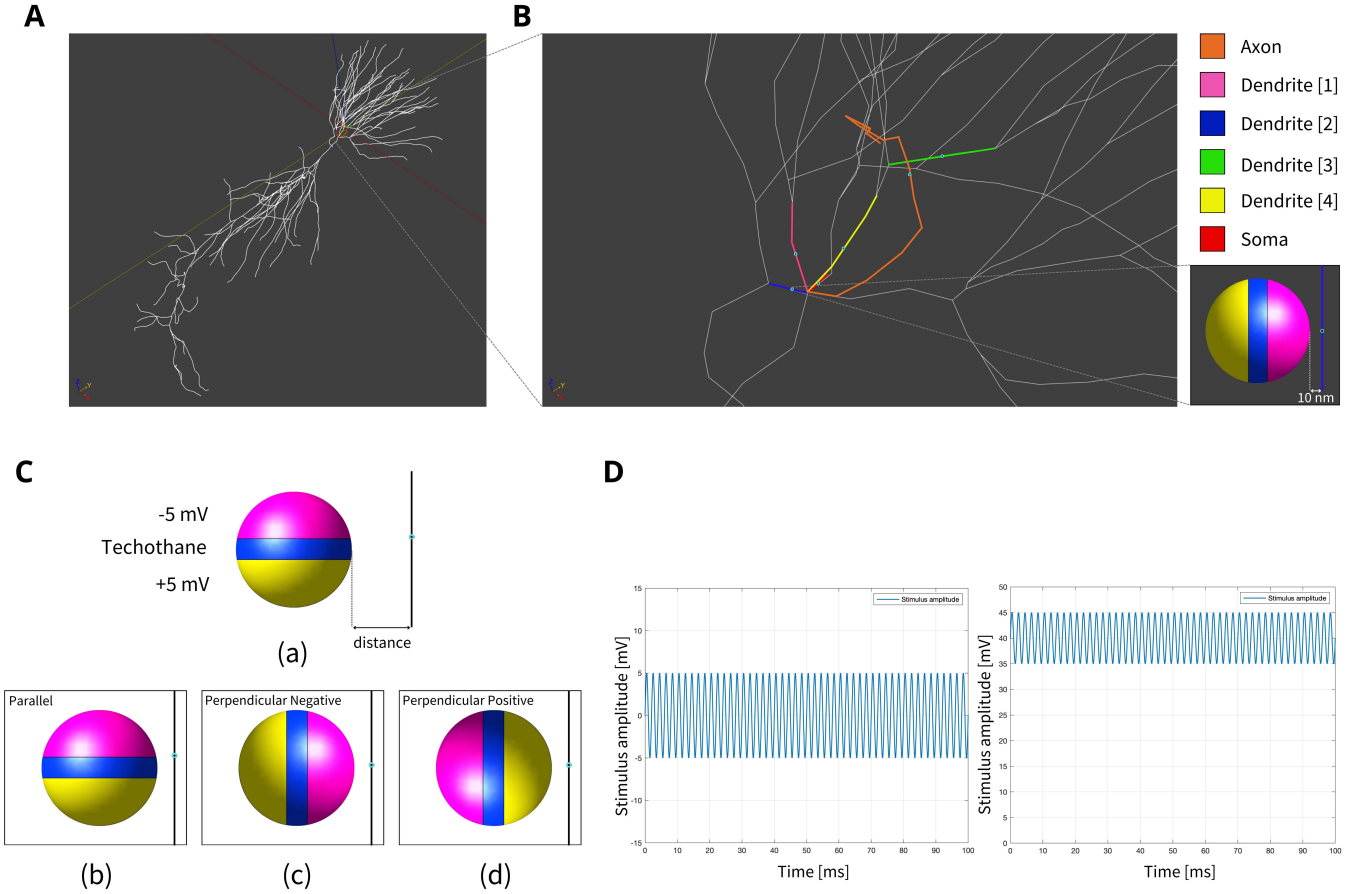
**(A)** Model of hippocampus CA1 neuron in the simulation environment. **(B)** A closer view of the neural compartments (highlighted in different colors) selected as targets for the placement of the MENPs, with zoom-in on the MENP's configuration close to the node of each compartment. The light blue dots displayed at the center of each segment represent the respective nodes. **(C)** Schematic representation of MENP model and configurations localized in proximity of the axon's node; (a) the MENP – node distance is defined as the distance between the node and the nearest edge of the dipole; (b) Parallel; (c) Perpendicular Negative; (d) Perpendicular Positive orientations. **(D)** Visualization of the sinusoidal waveforms of potential produced by each MENP applied in the simulations for the investigation of summation effects, featuring a frequency of 500 Hz, duration 100 ms and amplitude 5 mV: (left) without bias in the electric potential; (right) with a bias of 40 mV in the electric potential.

Due to the ME effect, upon exposure to a low-amplitude magnetic field, the surface of the MENP displays an electric potential with dipolar distribution that aligns with the direction of the external stimulating magnetic field (Betal et al., 2016). Drawing upon the findings from our prior multiphysics research (Fiocchi et al., 2022a; Marrella et al., 2023) and using an approach already employed in Galletta et al. (2024) and Chiaramello et al. (2022) for peripheral nerve stimulation and DBS, the electrical representation of the core-shell CoFe_2_O_4_-BaTiO_3_ composites was characterized by a dipolar configuration aligned with the presumed direction of the low-frequency magnetic field employed to induce the ME effect. Therefore, the ME effect was modeled by representing the geometric structure of the MENP as a sphere with two conductive surfaces possessing uniform potential, representing the peak amplitude achieved by MENPs when subjected to a uniform external magnetic field above magnetic saturation, i.e., a positive and a negative hemisphere of ± 5 mV, separated by an insulating layer consisting of Techothane material (V = 0 mV, σ = 0 S/m, ε = 3.4, and μ_r_ = 1) [Figure 1C (a)]. The selection of Techothane is based on an abstraction for modeling the dipole behavior, where any insulating material with negligible conductivity (σ ≈ 0) would be suitable. Although this model relies on a strong approximation, given the relative size of the neuronal cell and the MENPs, the spatial heterogeneity of the electric field distributions generated by real MENPs does not significantly influence the overall effect. This assumption is also supported by previous studies, such as Fiocchi et al. (2022a). ±5 mV was selected as a representative value based on the average values observed in previous studies (Zhang et al., 2022; Nguyen et al., 2021; Kozielski et al., 2021; Fiocchi et al., 2022b). While Marrella et al. (2023) report higher values, ±5 mV remains a plausible magnitude when considering experimental variability, controllability, and the broad range of α values documented in the literature. The choice of these boundary conditions is based on its suitability in emulating the macroscopic electric behavior of MENPs placed into a uniform AC magnetic field, with amplitude above magnetic saturation and assuming that they experienced the maximum polarization. The tissue medium was modeled as a 1 × 1 × 1.5 mm^3^ homogeneous and isotropic block with electric conductivity of 0.333 S/m, a representative value for the brain's gray matter (Butson and McIntyre, 2005; Saturnino et al., 2019). The EM simulation was set to low-frequency values of the AC magnetic field, in line with the range established for neural stimulation in literature (Pardo and Khizroev, 2022).

To account for the fact that MENPs are present in concentrations higher than that of a single MENP within the tissue, the entire volume occupied by many MENPs was hypothesized to be modeled as a dipole-shaped micrometric cluster. The dipole approximation used in our study aligns with the approach outlined in Chiaramello et al. (2022), ensuring consistency with existing methodologies. Specifically, hypothesizing that all the MENPs could be represented by dipoles orientated along the same direction, the approach used in the study follows the approximation of electric potential on the surface of a volume containing electric sources modeled as dipoles, based on the definition of “volume dipole moment density function” (Malmivuo and Plonsey, 1995).

At low frequencies, the EM ohmic quasi-static approximation applies and it was adopted to solve the EM problem, implementing a FEM numerical method previously employed and validated in similar studies for the solution of partial differential equations in complex geometries (Bayda et al., 2019; Fiocchi et al., 2022a; Samoudi et al., 2017); this solver can be used in electro quasi-static simulations when the ohmic current dominates the displacement current (Samoudi et al., 2017). The boundary settings were defined using Dirichlet conditions, where the potential is held constant at the poles of the spherical dipole, while electric fields and current densities are set to zero outside the simulation domain that encompasses the brain tissue.

It is important to note that under these stimulation conditions, MENPs operate off-resonance, effectively minimizing potential concerns regarding Joule heating effects. Computational estimations (Marrella et al., 2024) indicate that any temperature increase remains highly localized around the nanoparticle and rapidly dissipates with distance. Moreover, even minimal temperature changes may modulate neuronal ion channel activity, potentially influencing neuron excitability (Jabbari and Karamati, 2022; Van Hook, 2020). Nevertheless, it is well established that this effect does not compromise the safety of the approach in terms of cell viability and tissue integrity (Nguyen et al., 2021; Kozielski et al., 2021; Hadjikhani et al., 2017).

The grid optimization was achieved by manually adjusting the step size, setting a maximum of 1–6 mm for the space surrounding the MENP and the targeted neuronal segment, and 0.01 mm for the remaining brain tissue. This configuration resulted in a model meshed with approximately 30,000 M cells.

### 2.2 Neuron solver and neuron model

Sim4Life uses Neuron solver integrated libraries to assess the effects of induced EM fields on neuronal dynamics, by providing the option to directly integrate the outcomes of EM simulations with the neuronal dynamics solver. NEURON (Carnevale and Hines, 2006) is a simulation environment used for building, managing, and using computational models of neurons.

When subjected to an EM field, a neuron responds by altering its membrane's electrical activity, leading to localized and temporary variations in transmembrane potential (Malmivuo and Plonsey, 1995). The presence of inhomogeneity and swift fluctuations in the EM field can create pronounced localized potential gradients along the neuron, potentially depolarizing the membrane by driving the transmembrane flow of ionic currents (Malmivuo and Plonsey, 1995). If these changes exceed a certain threshold, they can trigger the generation of a spike or AP (Malmivuo and Plonsey, 1995). The initiation and propagation of APs is contingent on various factors, with the neuron's geometric arrangement and orientation relative to the EM field, as well as the specific transmembrane mechanisms, serving as crucial elements that determine the reaction to EM exposure (Malmivuo and Plonsey, 1995). A neuron exposed to an EM field senses electric potential (V) distribution; the electric field (E) is defined as the negative of the gradient of the potential:


(1) $$\begin{array}{l} \text{E}=-\text{ΔV} \end{array}$$

and thus


(2) $$\begin{array}{l} V=\;-\int_{l}\left(E\cdot dl\right) \end{array}$$

where *dl* is the path length integral (Hescham et al., 2015), and V is the distribution needed to run the Neuron solver.

The neuron model, selected from Model DB, a public database allowing for the use of realistic and specialized models (McDougal et al., 2016) (accession number 55035) and imported in Sim4Life, included the morphology, the mechanisms of neuronal dynamics, and the main biophysical elements responsible for spikes generation, and had already been validated against several experimental findings on electrophysiological and synaptic integration properties of CA1 neurons (Migliore et al., 2005). For what concerns the morphology, the modeled neuron consisted of a total of 178 sections, i.e., unbranched lengths of continuous cable connected, featuring precisely a soma, an axon, basal dendrites, apical dendrites, small ramifications or intermediate compartments, and a long section connecting the basal side of the neuron with the apical one. A representation of the neuron model imported in the simulation environment is provided in Figure 1A. Each section was discretized into two segments of equal length, displaying the section centers (nodes) at half of their length, that represented the points where all the biophysical properties of each section were concentrated for numerical simulation purposes. The biophysical properties were based on the Hodgkin-Huxley model, and the Neuron solver in Sim4Life computed the results by implementing the solution of the equivalent circuit using the cable equations that govern neuronal cells. The membranes were assigned uniform and standardized passive properties, including a membrane time constant (τ_m_) of 28 ms, membrane resistance (Rm) of 28 kΩ·cm^2^, axial resistance (Ra) of 150 Ω·cm, and a resting membrane potential (V_rest_) of −65 mV, while the core set of active properties encompassed sodium (Na), delayed rectifier potassium (KDR), A-type potassium (KA) conductances, and a mixed Na/K current activated by membrane hyperpolarization (I*h* current). The depolarization threshold was established at 80 mV, as done in similar applications in literature (Samoudi et al., 2017).

Within the Neuron dynamics solver, the titration mechanism was incorporated consistently across all simulations. Titration involves iteratively varying the stimulus intensity to determine the threshold at which the neuron undergoes sufficient depolarization to generate an AP. The equation governing the final threshold voltage [V_T_ (t)] is given by:


(3) $$\begin{array}{l} V_{T}\left(t\right)=V\;\cdot AC\cdot a\left(t\right) \end{array}$$

where V represents the static potential obtained from the EM simulation, *a(t)* is the modulating pulse with normalized amplitude modulating the potential V, and *AC* is the Amplification Coefficient, a unitless number whose value is adjusted until an AP is detected (Samoudi et al., 2017). An *AC* value > 1 indicates that a stronger stimulus is required to activate the neuron, whereas the optimal *AC* should be ≤ 1 to ensure efficient neuronal activation with the provided stimulus. Therefore, the AC serves as an indirect measure of the output voltage required for stimulation. In practical applications this voltage can be increased either by enhancing the external source field or by using MENPs with higher ME coefficient α. In an experimental setup, the AC would depend on multiple factors, including stimulation distance, MENP-to-axon distance, and field orientation.

The electric behavior of MENPs when placed into a uniform *AC* magnetic field was modeled as one period-sinusoids at a frequency of 100 Hz, similar to the typical repetition frequency used in DBS applications (Herrington et al., 2016), and amplitude 5 mV, given by *V* ^*^a(t), as reported in Equation 3 (Section 2.2).

### 2.3 Configurations and data analysis

The stimulation conditions considered for the purpose of the study can be classified into two categories: (1) axonal stimulation achieved with a single nanometric MENP and with a micrometric cluster modeling higher concentration of MENPs, separately, and (2) neuronal stimulation achieved through the placement of multiple MENPs at different neuronal targets to obtain neural summation effects. In all simulations, the positioning of the MENP was related to the center (i.e., node) of the target neural segment, and the distance was defined as the gap between the node and the nearest edge of the dipole [Figure 1C (a)].

#### 2.3.1 Single nanometric MENP and micrometric MENPs cluster

First, to achieve a very punctual and localized stimulation of the axon, the simulations were conducted by placing a single MENP with diameter of 100 nm close to the axonal segment. This choice aligns with sizes reported in the literature for MENPs-based stimulation, where diameters are typically adjustable within a range from a few tens of nanometers to over 200 nanometers (Pardo and Khizroev, 2022; Fiocchi et al., 2022b). Then, in the attempt to move toward a more realistic scenario, a micrometric cluster modeling the presence of a higher concentration of MENPs in a certain volume was used as electric source. Also this condition, considering comparable prior studies (Chiaramello et al., 2022), was computationally modeled following the same approach employed for the single nanoparticle, with the only variation being in the dimension of the dipole, as a diameter of 10 μm was chosen.

In both cases, three different orientations of the MENP with respect to the axonal segment were modeled and analyzed [Figure 1C (b–d)]: Parallel, featuring the axon's node aligned with the boundary separating the negatively charged pole and the dielectric layer; Perpendicular Negative, where the vertex of the negative pole was directed toward the axon's node; Perpendicular Positive, with the vertex of the positive pole oriented toward the axon's node. The influence of distance was examined for all the orientations, with the single MENP placed at 0.05 μm and 0.1 μm from the axon, and the cluster placed at distances of 5 μm and 10 μm, also denoted as 1r and 2r, corresponding to the radius and twice the radius, respectively, of the MENP and the cluster. In practical applications, these parameters can be modulated by adjusting the orientation of the magnetic field source (e.g., coils).

The analysis of the results was conducted using a set of indicators to ensure comprehensive evaluation. Initially, the distribution of the extracellular potential (V) over the axon's length was obtained, in pursuit of quantifying and comparing the EM stimuli delivered to the axon by the MENPs in different configurations. This is a key aspect for bridging the gap between the localized stimulation carried out by MENPs and the theory governing the effect of extracellular stimuli on neural membranes, which has been originally described by McNeal (1976), Rattay (1986), and Rattay (1998). To this extent, the second spatial derivative of V along the axon was computed, since this metric is identified with the activating function (AF) (Equation 4), a concept introduced by Rattay (1986), that has been applied for decades to predict the behavior of neurons in response to extracellular stimulation (Aplin and Fridman, 2019).


(4) $$\begin{array}{l} AF\left(x\right)=\frac{\partial^{2}V\left(x\right)}{\partial x^{2}} \end{array}$$

where *x* is the axon's length and V(*x*) is the value of external potential sensed at *x*. According to the classical cable theory, long, and straight neural segments can experience activation or inhibition depending on the polarity of the AF (Rattay, 1998). In the framework of the current research, the AF was quantified only for stimulation with the nanometric MENP, since in this condition the EM distributions were extremely localized over a short range, allowing for the approximation of the axon as a long and straight segment. Conversely, the AF was not derived in the case of the cluster since the extended potential distribution around the cluster and along the axon also affects the bended sections; consequently, the approximation of the axon as a long and straight segment is not allowed, and any correlation between the second derivative along a straight line and neuron activation is invalidated.

Afterwards, the amplification coefficient served as the most valuable indicator for estimating the required stimulus amplitude conveyed by the MENP to evoke an AP in the neuron.

#### 2.3.2 Summation effect with multiple MENPs (100 nm)

Following the initial set of simulations, the study investigated the summation effects generated by multiple MENPs positioned near the axon and distributed across the adjacent basal dendritic arbor. Various configurations were modeled, targeting distinct combinations of neuronal compartments, including the axon, soma, and four dendrites. Individual MENPs were precisely placed within specific segments, oriented according to the Perpendicular Negative configuration (described in Section 2.3.1) and maintained at a constant distance of 10 nm from their respective targets. This orientation was chosen based on its performance with respect to the other orientations in the previous simulations. Figure 1B illustrates the neural compartments selected for MENPs' placement and the configuration designed to evaluate summation effects. Sinusoidal stimuli with a fixed duration of 100 ms and amplitude of 5 mV were applied, varying in frequency and value of static potential bias. When discussing the involvement of one, two, three or four dendrites, this refers to the testing of multiple combinations of neuronal compartments involving the specified number of dendrites. For instance, when targeting two dendrites, different pairings, such as dendrite 1 + dendrite 2, dendrite 2 + dendrite 3, and so on, were analyzed. This systematic approach ensured a comprehensive evaluation of the potential effects of MENPs across diverse spatial configurations.

Given that neuronal cells exhibit frequency-dependent behavior, our study of summation effects included the neuron response to variations in the frequency of the modulating stimulus. Initially, three MENPs were modeled and positioned around the neuron to target three distinct segments: the axon and two dendrites. The applied stimulus consisted of a sinusoidal waveform, with frequencies ranging from 50 Hz to 10 kHz. Following these results, a fixed sinusoidal frequency of 500 Hz was used to target seven different segment combinations: axon + one dendrite, axon + two dendrites, axon + three dendrites, axon + four dendrites, axon + three dendrites + soma, axon + four dendrites + soma, and four dendrites + soma.

To further refine the analysis and identify the optimal combination of target configurations and stimulus parameters capable of activating the neuron with AC ≤ 1, a static bias was introduced while maintaining the 500 Hz sinusoidal frequency. This bias simulates the hypothetical modulation of the external DC magnetic field in conjunction with the ME coupling properties of the MENPs. The bias value was varied incrementally between 5 mV and 40 mV in 5 mV steps. Under these conditions, three target configurations were evaluated: axon + two dendrites, axon + three dendrites + soma, and axon + four dendrites + soma. The sinusoidal waveforms of the potential produced by MENPs, both without bias and with a 40 mV bias, are depicted in Figure 1D.

The results were assessed based on several factors, including the amplification coefficient required to elicit neuronal activation, the axon's membrane potential (V_m_), the location of the initial spike, and its propagation across the neuron's compartments.

## 3 Results

### 3.1 Single nanometric MENP and micrometric MENPs cluster

**Figure 2:**
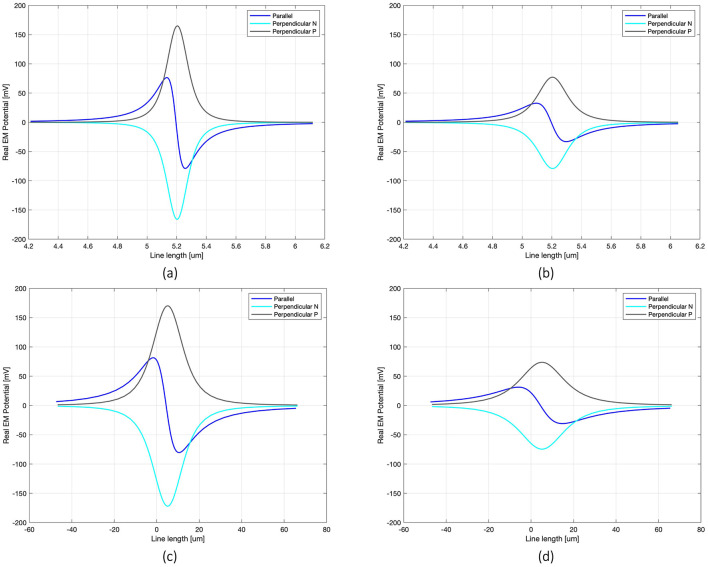
Real V distribution along the line passing through the axon for each modeled configuration: 100 nm single MENP placed at distance 0.05 μm **(a)** and 0.1 μm **(b)** from the axon, with focus on 1.5 μm range around the node; cluster at distance 5 μm **(c)** and 10 μm **(d)** from the axon, considering 100 μm range around the node.

This section presents the outcomes relative to the electric potential (V) distribution along the axon, the amplification coefficient for an axonal initial spike, and the axon's membrane potential with electric sources consisting of the 100 nm diameter single MENP and the 10 μm diameter cluster, evaluated separately. Specifically, for both the nanometric MENP and the micrometric cluster, a comparison between three different orientations at two distinct distances between the electric source and the axon is presented. Figure 2 shows the distributions along the axon of the real part of V when varying the MENP's size, orientation and distance relative to the axon. For clarity, the plots of the distributions focus on the central section of the axon, zooming in on the region around the node. The zoomed-in ranges are 4.2–6.2 μm for the nanometric MENP (Figures 2a, b) and −45–60 μm for the cluster (Figures 2c, d), which encompass the main effect of the EM stimulation delivered to the axon. The axon's node is located exactly at the x-axis coordinate of 5.2 μm.

The V distributions along the axon highlight the significant impact of MENP's orientation on the transmission of EM signals when the distance between the MENP and the axon is held constant. The shape of the V distribution, which is determined by the relative positioning of the positive and negative poles with respect to the axon, allows for classification of the stimulus as cathodic (Perpendicular Negative), anodic (Perpendicular Positive), or bipolar (Parallel) stimulation. The Perpendicular orientation resulted in the highest peak V amplitude (0.16 V), while the Parallel orientation produced approximately half of the maximum peak. As expected, increasing the distance between the MENP and the axon led to a consistent reduction in V, along with a slight broadening of the distribution curves. As the number of MENPs increased, modeled as a cluster, a comparison of the x-axis values in Figures 2a–d reveals a pronounced spreading of the V distribution range along the axon.

**Figure 3:**
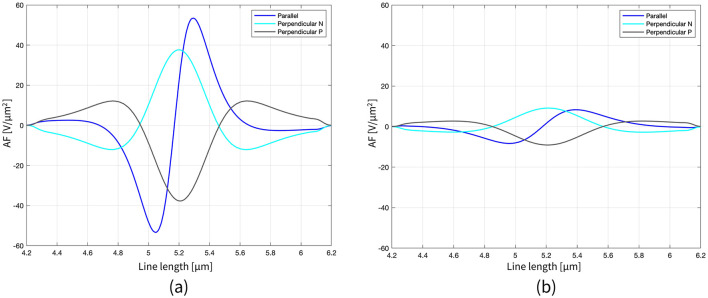
Representation of the AF along the axon length (2 μm range around the node) for all the configurations, when the distance single MENP-axon is 0.05 μm **(a)** and 0.1 μm **(b)**.

Moving to the activating function (AF), namely the second spatial derivative of the potential along the axon, Figure 3 shows its behavior for the three orientations and two distances simulated in the case of the nanometric MENP. Similarly to the V distribution plots, the computation of the AF was limited to a narrow range around the axonal node. As expected, the “anodic” orientation displays a negative peak at the center, indicating hyperpolarization, with positive lobes on either side producing depolarization. Conversely, the “cathodic” orientation exhibits prominent depolarization at the center, with minor hyperpolarization at the sides. The “biphasic” configuration, on the other hand, features a sequence of hyperpolarization followed by depolarization. Again, increasing the distance between the MENP and the axon translates into reduced AF values and broader distributions along the axon.

Table 1 summarizes the amplification coefficient values obtained from both the single MENP and cluster models for the simulated configurations. The designation “no axon stimulation” for the Perpendicular Positive orientation indicates that a spike was initiated in the neuron but remained confined to a dendritic branch, without propagating to the axon or any other neuron compartment. In contrast, the cluster in the Parallel orientation at a distance of 2r successfully triggered an AP in a dendrite, which then propagated to activate all basal dendritic branches and the axon.

**Table 1:** Values of amplification coefficient for single MENP and MENPs cluster for each modeled configuration.

**Dimension**	**Orientation**	**Orientation view**	**Amplification coefficient**	
			**1r**	**2r**
Nanometric MENP	Parallel	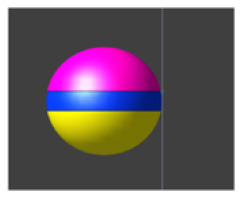	172	189
	Perpendicular negative	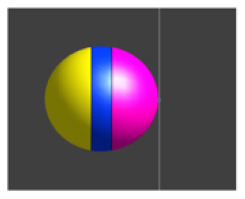	756	525
				
	Perpendicular positive	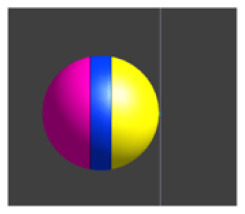	No axon stimulation	No axon stimulation
Micrometric cluster	Parallel	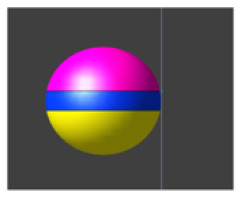	261	Starting from dendrite
	Perpendicular negative	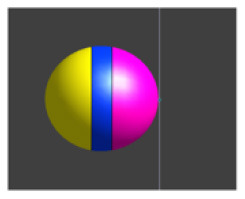	14	29
				
	Perpendicular positive	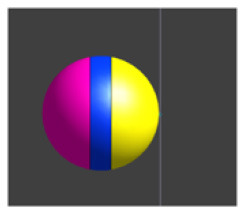	No axon stimulation	No axon stimulation

### 3.2 Summation effect with multiple nanometric MENPs on dendrites

This section presents the results of integrating stimuli provided by multiple MENPs distributed across the dendritic tree (Figure 1B), with varying targets and modulating waveforms. As described in Section 2.3.2, various combinations of neuronal compartments, each involving a specific number of dendrites, were evaluated. The results demonstrated that the particular selection of dendrites within a given group (e.g., specific pairs or triplets of dendrites) did not influence the observed outcomes. This suggests that the summation effects were not specific to individual dendritic compartments but were instead dependent on the total number of dendrites targeted. Consequently, some of the subsequent results are presented without reference to specific dendritic combinations, focusing instead on the number of dendrites targeted.

**Figure 4:**
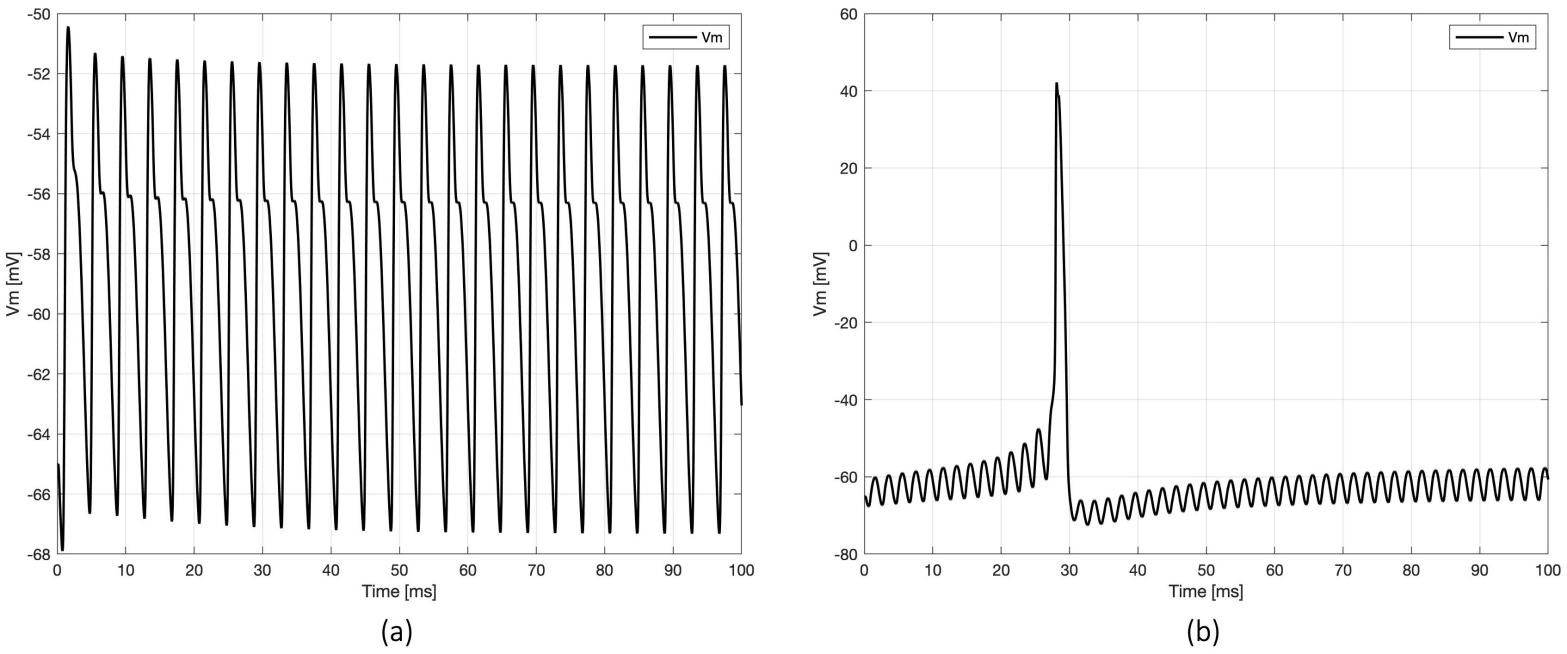
Examples of membrane potential (*Vm*) dynamics registered at the axon's node under two scenarios of neuronal activation: **(a)** spike initiation in the dendrites, with no propagation to the axon (at frequency 250 Hz), and **(b)** spike initiation in the axon (at frequency 500 Hz).

The outputs from simulations examining the summation effects obtained with three MENPs—one placed close to the axon and the other two positioned near distinct dendrites—under different sinusoidal frequencies are detailed in Table 2. The table provides the amplification coefficients necessary to trigger neuronal activation across frequencies ranging from 50 Hz to 10 kHz. Notably, the location of the initial spike varied depending on the frequency. At frequencies up to 250 Hz and above 10 kHz, a local spike raised in one of the dendrites but did not propagate to the axon or any other compartment of the neuron. In contrast, for intermediate frequencies, signals originating from multiple neuronal targets were effectively integrated, leading to spike initiation in the axon. To illustrate these dynamics, Figure 4 presents representative plots of the axon's membrane potential (V_m_) in both scenarios.

**Table 2:** Amplification coefficients required to elicit neuronal activation, and neuronal location of the first spike under varying stimulus frequencies, following stimulation with one MENP close to the axon and two near distinct dendrites.

**Sinusoidal frequency**	**50 Hz**	**100 Hz**	**250 Hz**	**400 Hz**	**500 Hz**	**600 Hz**	**750 Hz**	**1 kHz**	**10 kHz**
Location of first spike	Dend	Dend	Dend	Axon	Axon	Axon	Axon	Axon	Dend
Amplification coefficient	31	18.9	14.4	10.2	10.6	10.9	11.5	12.3	33.3

Based on the previous results, which suggest that the range [400–600 Hz] is the most promising frequency for spike arising, the intermediate frequency of 500 Hz was selected for all subsequent simulations. Notably, stimulation with two MENPs—one placed on the axon and the other on a dendrite—failed to activate the neuron. Activation was achieved with relatively low amplification coefficients by placing additional MENPs on other dendrites and the soma (Table 3). Specifically, the tested configurations included axon + two dendrites, axon + three dendrites, and axon + four dendrites, with amplification coefficients ranging from 10.56 to 11. The configurations axon + three dendrites + soma, axon + four dendrites + soma, and four dendrites + soma produced coefficients between 11 and 11.8. In all these stimulation settings, the AP was generated in the axon, with the V_m_ closely resembling that shown in Figure 4b, and subsequently back-propagated throughout the neuron. The plots in Figure 4 highlight variations in axon V_m_ profiles and propagation patterns, underscoring the influence of the stimulus-dependent summation effects on neuronal activation.

**Table 3:** Amplification coefficients required to elicit neuronal activation for local stimulation of different combinations of neuronal segments, when applying a 500 Hz sinusoidal stimulus.

**Neuronal segments combination**	**Axon + 2 dendrites**	**Axon + 3 dendrites**	**Axon + 4 dendrites**	**Axon + 3 dendrites + soma**	**Axon + 4 dendrites + soma**	**4 dendrites + soma**
Amplification coefficient	10.56	10.56	11.0	11.0	11.5	11.8

**Figure 5:**
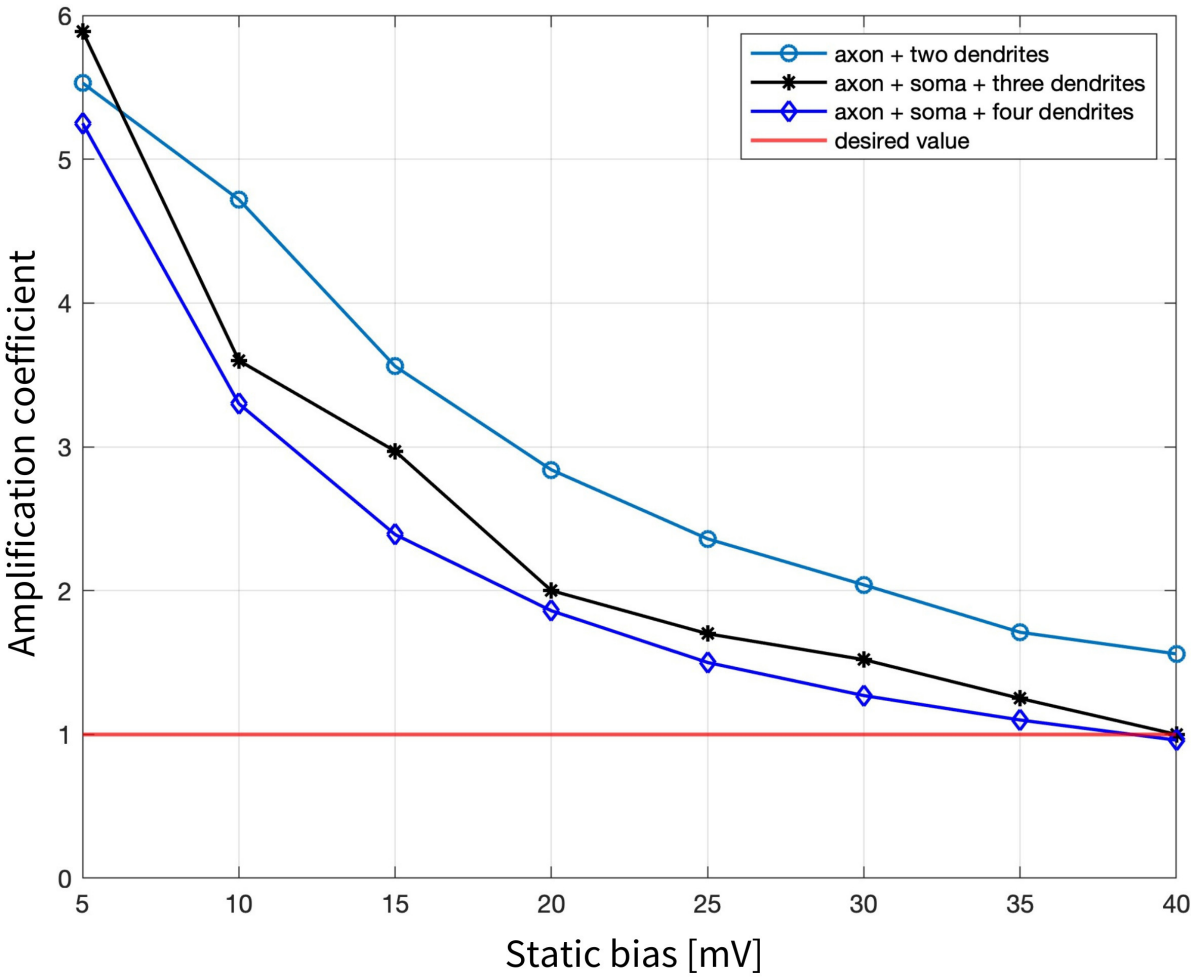
Amplification coefficients required to elicit neuronal activation for local stimulation of the three tested combinations: axon + two dendrites, axon + soma + three dendrites, and axon + soma + four dendrites, when changing the static bias applied to the 500 Hz sinusoidal stimulus from 5 mV to 40 mV.

Building on these findings, while maintaining a constant sinusoidal frequency of 500 Hz, a static bias in the electric potential generated by the MENPs was introduced. Three target combinations were tested: axon + two dendrites, axon + soma + three dendrites, and axon + soma + four dendrites (all the compartments shown in Figure 1B), and the respective results are presented and compared in Figure 5. In all cases, the amplification coefficient gradually declined as the bias increased, starting at approximately 5 for a bias of 5 mV and declining to below 1 at a bias of 40 mV. However, a notable difference was observed between the configurations: the combination with two dendrites resulted in localized activation confined to a single dendrite when the bias exceeded 5 mV (displaying an axon's V_m_ similar to Figure 4a), whereas the other two combinations elicited a spike in the axon that back-propagated to other compartments, irrespective of the bias value (same V_m_ pattern shown in Figure 4b).

## 4 Discussion

The growing demand for novel techniques in electrical stimulation of the central nervous system has drawn attention to the potential of magnetoelectric nanoparticles (MENPs) as a promising tool for wireless and minimally invasive stimulation of specific deep brain regions, all while maintaining good biocompatibility (Nguyen et al., 2021; Kozielski et al., 2021). This approach could pave the way for advanced treatments in neurodegenerative diseases, as highlighted in previous studies (Khizroev, 2018). Computational methods play a critical role in evaluating MENPs' interactions with brain tissues, enabling the identification of key factors that could optimize their transition to clinical applications. This study aligns with this goal, offering a numerical approach to assess the feasibility of MENPs for evoking neural responses. Specifically, we focused on the effects of different MENP configurations—including concentration, orientation, distance, and placement—as well as modulating signals (frequency and amplitude) on stimulating a hippocampus CA1 pyramidal neuron model. Several metrics were evaluated, including local electric potential distributions, activating function, stimulus amplitude required to evoke APs, spike propagation and axonal membrane potential. The findings offer important insights into the factors influencing the effectiveness of MENP-mediated neural stimulation.

The first set of simulations, designed to locally stimulate the axon, demonstrated the feasibility of precise stimulation with MENPs, enabling exceptionally high spatial resolution. The results proved that the presence of higher concentrations of MENPs, modeled as a micrometric cluster, extended the spatial range of the stimulus, enhancing the efficiency of excitation by spreading the potential over a larger area. This was reflected in the electric potential distributions and lower amplification coefficients (Figure 2; Table 1). Furthermore, the orientation of the MENPs emerged as a critical determinant shaping the stimulation outcomes. The electric potential distribution along the axon was significantly impacted by the MENP's orientation, with the perpendicular configurations yielding peak amplitudes nearly double those of the parallel orientation. This finding emphasizes the ability of perpendicular orientations to generate stronger localized potentials along the axonal membrane (Figure 2). The impact of orientation on the stimulation outcomes is linked to opening and closing of ion channels in the immediate vicinity of the nanoparticle (Zhang et al., 2024). The orientation also plays a crucial role in shaping the activating function dynamics. The different shapes of the activating function for each orientation correspond to variations in the regions of depolarization and hyperpolarization along the axon, both in terms of amplitude and spatial extent (Figure 3). Specifically, parallel configurations, which resemble bipolar electrode stimulation, are more likely to trigger APs than perpendicular configurations when MENPs are placed very close to the axon. This result suggests that the orientation impacts the minimum stimulus needed to activate the neuron (Table 1). While the parallel orientation demonstrated lower amplification coefficients for single MENPs compared to the perpendicular negative configuration, the trend reversed when higher concentrations of MENPs were used, highlighting the concentration-dependent interplay between MENP configuration and stimulation efficiency. This is likely due to the influence of different distributions of electric potential resulting from different MENPs configurations on spiking probability. In contrast, the perpendicular positive orientation, resembling cathodal stimulation, consistently failed to induce neuronal activation, indicating its limited efficacy in depolarizing the neuronal membrane. As expected, the distance between the MENP and the axon negatively affected both the electric potential distributions and the activating function, leading to a reduction in values and widening of the curves as the distance increased. This attenuation in the stimulus was also reflected in the amplification coefficient values, which indicated a significant reduction in the efficacy of stimulation as the distance between the MENPs and the axon increases. These results suggest that optimizing the orientation, while minimizing the distance between MENPs and the axon, is crucial for achieving effective localized stimulation. Indeed, other studies support that, to enhance stimulation efficacy, nanoparticles should ideally be positioned with their poles in direct contact with the membrane surface (Zhang et al., 2022). Therefore, our approach is conservative in this regard, as we position the MENPs at distances of 1r and 2r.

Following these initial considerations, the study delved into the summation effects of multiple MENPs strategically positioned across the basal dendritic tree adjacent to the axon, to emulate the physiological convergence of signals within the dendritic arbor that facilitates AP generation at the axonal level, using an approach of subthreshold stimulation similar to previous studies (Kim et al., 2023), and exploiting evidence of integration of local depolarization of dendritic membranes driven by MENPs (Zhang et al., 2022). The findings highlighted the interplay between spatial arrangement of MENPs and modulating stimulus in shaping neuronal responses and proved the ability of multiple MENPs to successfully activate the neuron. Simulations with different combinations of neuronal compartments comprising a set number of dendrites revealed that the selection of specific dendrites within a given group (e.g., particular pairs or triplets of dendrites) did not notably influence the outcomes. This insight indicates that summation effects are not tied to singular dendritic compartments, and helps establish the reliability of the observed summation effects by ensuring that the findings are broadly applicable to similar configurations. This observation is consistent with prior research, which suggests that synaptic integration in neurons is largely independent of input location, likely due to the compensatory mechanisms that counteract the intrinsic filtering properties of dendrites (Magee, 2000). Along with that, the number of neuronal segments stimulated appeared to exert minimal impact on the activation outcomes, as configurations involving three or more segments showed consistent activation patterns across all tested configurations. Specifically, an amplification coefficient of around 11 was sufficient to generate an AP in the axon, which subsequently back-propagated through the neuron. This consistency suggests that a robust integration mechanism is in place, one that is relatively insensitive to the specific arrangement of the stimulated segments. However, the two-segment configuration did not produce activation, indicating that the involvement of at least three neuronal segments is necessary for successful neuronal stimulation under these conditions. This observation is consistent with previous evidence showing the necessity of engaging multiple neuronal compartments for effective stimulation (Tomko et al., 2021). Additionally, the stimulation of the axon combined with two dendrites exhibited sensitivity to the waveform shape of the applied stimulus when an electric bias was introduced. In practical applications, exploiting summation effects for stimulation may raise concerns about the spatial summation of electric fields. However, despite the challenge of controlling the induced electric fields, the relatively small size of MENPs and their spacing, coupled with the rapid decay of the electric field with distance, makes direct field summation negligible.

Knowing that neuronal cells exhibit frequency-dependent behavior, the effect of stimulation frequency was also investigated, shedding light on its role in spike initiation and propagation. Low-frequency stimuli (up to 250 Hz) and very high-frequency stimuli (10 kHz) elicited localized APs confined to the dendrites, without sufficient integration for propagation to the axon (Figure 4). These localized responses were associated with higher amplification coefficients, as shown in Table 2, indicating that stronger stimuli were required for even partial activation. The described behavior aligns with evidence from prior studies on CA1 pyramidal neurons, which indicate that the transmission of dendritic APs to the soma and axon is inherently limited (Gasparini et al., 2004) and the likelihood of propagation might hinge upon factors such as changes in the membrane potential, distance between the initiation site and soma, and stimulus frequency (Gasparini et al., 2004). At lower frequencies, failure to sustain depolarization beyond the threshold for axonal spiking is likely due to insufficient temporal summation, as low-frequency inputs, with their longer intervals, might allow excitatory postsynaptic potentials to decay before reaching the threshold for spike initiation. Indeed, studies support that dendrites, characterized by lower excitability than axons, require large and fast synaptic potentials to generate spikes (Spruston et al., 2016). Empirical evidence further supports that frequencies above 100 Hz are generally more effective, as seen in deep brain stimulation (DBS) (Herrington et al., 2016). On the other hand, kilohertz-frequencies can induce a neural conduction block by inhibiting AP propagation through the axon (Neudorfer et al., 2021). It is relevant to clarify that, although resonance effects could significantly enhance the electric output of MENPs, our approach intentionally operates off-resonance. The primary reason for this choice is that we aim to position MENPs directly at the interface with neuronal cells, without any additional electric interface. At nanometric scales, MENPs resonate at frequencies in the GHz range, while, even increasing their size to the micrometric scale, the resonance frequency shifts to the MHz range (Singer et al., 2020; Chen et al., 2023). However, it is well established that effective neural tissue stimulation occurs below the kHz range. This constraint means that resonance-based mechanisms would require significantly larger MENPs, which may not be practical for direct neuronal interaction. Therefore, while MENP's output is undoubtedly different at resonance, the frequencies involved are too high to effectively interact with the nervous system. Another important consideration in off-resonant powering of the MENPs is the minimization of Joule heating effects, which are typically observed at higher frequencies. While a slight temperature increase may occur, it could actually enhance neuron stimulation by influencing ion channel activity (Jabbari and Karamati, 2022; Van Hook, 2020). However, this does not pose a safety concern regarding biocompatibility, as several experimental studies have demonstrated (Nguyen et al., 2021; Kozielski et al., 2021; Hadjikhani et al., 2017).

Finally, simulations were performed under the hypothesis that the modulation of the external DC magnetic field, in conjunction with the ME coupling properties of the MENPs, would generate a potential composed of a steady offset and an oscillatory component. This approach successfully triggered APs when the field intensity produced an electric potential bias >35 mV, as indicated by the amplification coefficient dropping below 1. These outcomes showed the effectiveness of multiple MENPs in achieving targeted neuronal modulation by leveraging the combined effect of static and oscillatory fields to reliably affect the activation threshold, a strategy previously demonstrated by other research groups (Kozielski et al., 2021).

Overall, the results of our study are consistent with and build upon the findings of previous research, offering additional insights into the underlying mechanisms and potential applications (Zhang et al., 2022, 2024; Kozielski et al., 2021; Herrington et al., 2016; Kim et al., 2023; Magee, 2000; Tomko et al., 2021; Gasparini et al., 2004; Spruston et al., 2016; Neudorfer et al., 2021).

It is important to acknowledge that the outcomes examined in this study are based on numerical simulations of extremely simplified conditions. In the first place, modeling MENPs as idealized spherical dipoles with fixed boundary potentials (±5 mV) is indeed a strong simplification. However, this serves as a reasonable first approximation of the ME effect, which follows an approach extensively validated in prior studies (Fiocchi et al., 2022a; Marrella et al., 2023; Galletta et al., 2024; Chiaramello et al., 2022). Regarding the model of the cluster, by assuming that all MENPs are aligned in the same direction, our approach adheres to the established approximation of electric potential on the surface of a volume containing electric sources modeled as dipoles, as defined by the definition of the “volume dipole moment (Malmivuo and Plonsey, 1995) density function.” Additionally, considering the relative sizes of neuronal cell and MENPs, in practical applications, spatial variations in the electric field are unlikely to significantly influence the overall effect (Fiocchi et al., 2022a). Nevertheless, certain parameters in real implementations remain beyond our control. While the orientation and the exact placement of MENPs can be manipulated by rotating the external magnetic field source and by functionalizing MENPs with specific antibodies or other molecules to target specific membrane receptors (Lee et al., 2023), respectively, other factors, such as the concentration of nanoparticles and the amplitude of generated electric potential, are subject to fluctuation due to variations in manufacturing processes. Hence, as a concluding remark and primary insight derived from this study, by optimizing MENPs' configuration, it is possible to achieve controlled and reproducible neuronal activation, offering a promising avenue for advancing the field of neurostimulation. However, it is essential to emphasize that the values of electromagnetic distributions required for neural activation are not fixed and must be tailored to specific stimulation conditions.

In would be interesting, in future investigations, to assess the potential of other configurations of nanoparticles, e.g., microsized structures operating at resonance, arrays of magnetoelectric structures to be positioned either on the skull or the scalp coupled with external magnetic field generators for greater distance and less invasive purposes, MENPs coated with hydrogels to improve the control, the stability, and the charge retention, enhancing in parallel the effectiveness and reliability of the technique.

In conclusion, this study demonstrates for the first time the viability of applying MENPs as innovative approach for precise electric stimulation of a hippocampus CA1 neuron, underscoring the critical role of MENPs' concentration, orientation, distance, location, modulating stimulus delivered, and shows great promise as a powerful tool for targeted neural modulation, with potential applications of such groundbreaking technology in the stimulation of deep neural tissues, opening up new avenues for the treatment of Alzheimer's disease and other neurodegenerative disorders.

## Data Availability

The raw data supporting the conclusions of this article will be made available by the authors, without undue reservation.

## Footnotes

^1^Sim4life by ZMT Zurich Med Tech AG, Zurich, Switzerland. Available online at: http://www.zurichmedtech.com.
